# A Microservices e-Health System for Ecological Frailty Assessment Using Wearables †

**DOI:** 10.3390/s20123427

**Published:** 2020-06-17

**Authors:** Francisco M. Garcia-Moreno, Maria Bermudez-Edo, José Luis Garrido, Estefanía Rodríguez-García, José Manuel Pérez-Mármol, María José Rodríguez-Fórtiz

**Affiliations:** 1 Department of Software Engineering, Computer Sciences School, University of Granada, 18014 Granada, Spain; mbe@ugr.es (M.B.-E.); jgarrido@ugr.es (J.L.G.); mjfortiz@ugr.es (M.J.R.-F.); 2 Department of Physiology, Faculty of Health Sciences, University of Granada, 18016 Granada, Spain; rodrigueze@ugr.es (E.R.-G.); josemapm@ugr.es (J.M.P.-M.)

**Keywords:** wearable devices, sensors, mobile health systems, microservices architecture, IoT, machine learning, elderly frailty assessment, e-health

## Abstract

The population in developed countries is aging and this fact results in high elderly health costs, as well as a decrease in the number of active working members to support these costs. This could lead to a collapse of the current systems. One of the first insights of the decline in elderly people is frailty, which could be decelerated if it is detected at an early stage. Nowadays, health professionals measure frailty manually through questionnaires and tests of strength or gait focused on the physical dimension. Sensors are increasingly used to measure and monitor different e-health indicators while the user is performing Basic Activities of Daily Life (BADL). In this paper, we present a system based on microservices architecture, which collects sensory data while the older adults perform Instrumental ADLs (IADLs) in combination with BADLs. IADLs involve physical dimension, but also cognitive and social dimensions. With the sensory data we built a machine learning model to assess frailty status which outperforms the previous works that only used BADLs. Our model is accurate, ecological, non-intrusive, flexible and can help health professionals to automatically detect frailty.

## 1. Introduction

In a global ageing society, it is important to detect the frailty in the early stages to be able to slow down the decline of elderly people and to keep them active and healthy for as long as possible. Frailty is a syndrome of older adults that increases the risk of falls, hospitalizations and even death [1,2]; affecting 11% of the home-dwelling elderly population without hospitalization and increasing drastically to 30–70% of the surgical patients over 65 years old [3]. The early detection of frailty has been proved to increase the independence of the older adults and the decrease on health care costs [4,5].

The current detection of frailty is performed manually via independent tests of strength, gait or self-report questionnaires, being Fried [4] the most used test, which only assess the physical dimension of frailty. Some authors have recently accepted that frailty involves not only the physical dimension, but also social and cognitive dimensions [6]. Accordingly, some wider tests have appeared which include the three dimensions of frailty, such as the Tilburg Frailty Indicator (TFI) test [7]. However, the tests are time consuming, and require investment in health resources and physical interaction between patients and doctors. Indeed, the detection and manual assessment of the frailty for all older people is not currently affordable, especially due to the organizational and financial issues mentioned above [8].

Frailty also affects the performance of the Instrumental Activities of Daily Living (IADLs) [9]. The Activities of Daily Living (ADL) are classified as basic (BADL), such as toilet hygiene or functional mobility; Instrumental (IADL), such as house cleaning or shopping; and advanced (AADL), such as travelling and social events. BADLs are crucial for the human being´s survival and they involve less-demanding activities in terms of physical or cognitive resources in comparison with IADLs. These activities are commonly named as self-activities performed by the person without the need of help or interaction with other people. On the other hand, IADLs and AADLs are more complex activities that involve more resources than BALDs in terms of physical, cognitive and social functions. These activities usually also involve the interaction with other objects or tools. Monitoring of the performance of the IADLs could potentially be a possible marker of the frailty status, since the complexity of the IADLs make elders them more vulnerable to show dependence in their performance. Therefore, the monitoring of the IADLs could replace the manual tests or questionnaires, alleviating the health financial and organizational requirements to globally asses frailty. This alleviation could be possible if the monitorization is performed with low-cost or well-spread devices, such as sensors, that are currently built-in in smartphones or wearables.

Wearable devices, together with data analysis techniques, can seamlessly monitor and assess the frailty status during the performance of the IADLs. This could be even less intrusive in the near future, because it is foreseen that the majority of the population, including elderly people, will wear wearables with built-in sensors [10], especially after the pandemic coronavirus of 2019 [11]. Sensors have been successfully used in several e-health scenarios [12]. They can provide efficient and precise psychophysiological and location values [13]. Some research works have started to measure frailty indicators [3,14,15,16,17,18], being the most used devices electronic stickers, bands, bracelets, smartphones and smartwatches [15,19,20,21,22,23]. However, these approaches only measure the physical dimension of the frailty during a BADL with only one sensor, either the accelerometer or the gyroscope. None of them monitored IADLs, such as a real shopping in the supermarket, which involves not only physical activity, but also cognitive and social interactions. Furthermore, existing approaches are also limited in terms of flexibility, extension and evolution, as they do not take advantage (at design and deployment levels) of software architectures that allow developers to easily integrate current and future sensors and functionalities. Hence, to the best of our knowledge, there is no flexible approach which assesses the frailty status through the monitoring of IADLs in an ecological way, i.e., in the daily living environment of older adults.

To overcome these limitations, we expanded our microservices architecture proposed in [24], with a choreography solution and a use case that collects sensory data from wearables and builds a Machine Learning (ML) model to assess frailty in an ecological manner—while the older adult is performing an IADL, in combination with BADLs. Service-Oriented Architectures (SOA) are well-known for its generalization, and therefore for its reuse in different scenarios. They can also deal with mobility, integration interoperability, fault tolerance and self-adaptation [25,26,27,28,29]. A microservices architecture improves the SOA approach, following the concept share-as-little-as-possible [30], with additional system characteristics such as decoupling, flexibility, extension, scalability and evolution [31,32]. Microservices architectures allow to collect a wide variety of sensory data under the Internet of Things (IoT) paradigm. We also explored several ML algorithms in order to build the most accurate model to assess frailty. In our proposal we use microservices for data collection, data analysis and to expose the frailty model as a microservice, with the aim of reusing the microservices in future use cases. 

Our proposal is ecological and effective in terms of costs and time-consuming reduction, using an accurate frailty assessment model that outperforms the related works. We collect sensory data in a non-intrusive and transparent manner while the older adult is performing the IADL of shopping; without disturbing neither the person daily life nor his environmental conditions (same supermarket, time, pathway, etc.), i.e., ecologically. Measuring the performance of IADL we can gain a comprehensive vision of elderly people disorders at physical, cognitive and/or social levels. Hence, health professionals can design specific interventional programs to improve the frailty, increasing the independence of the older adults and decreasing health care costs. Furthermore, with the microservices architecture, the system could be easily extended [33] to address other use cases, by adding new microservices with the corresponding ML algorithms, models and sensors. For example, we could add microservices for the ML models built for a different population, with specific pathologies, ethnicities; or we could add microservices for collecting data coming from new sensors, or data sources. Our results outperformed the ecological related works, which use only the physical data from the performing of BADLs (instead of IADLs) and are non-low-cost. These findings have important implications for healthcare systems, because our automatic frailty detection system can reduce health costs and health professionals time assessing frailty ecologically with low-cost devices.

The rest of the paper is organized as follows: Section 2 introduces the related works. Section 3 describes the design of the microservices system architecture to collect and analyze data, as well as a description of the data analysis pipeline. Section 4 presents a system validation and the results of the ML frailty model performance. Section 5 discusses the findings. And the last section summarizes conclusions and future work.

## 2. Related Work

In order to monitor a variety of sensory data, we need to integrate and combine several sensors through systems, which usually use IoT and/or microservices architectures [34,35,36]. IoT health systems allow doctors to monitor patients constantly and to suggest corrective actions according to the analyzed data [29,37,38]. For example, one of the goals of City-4Age and ACTIVAGE H2020 projects is the assessment of frailty using IoT technology based on rules [39]. Furthermore, there are a few efforts to apply microservices in the health domain. For example, some studies focused on security and privacy of microservices architectures for the sensible data in the health systems [40,41]; other work explored monitorization of depression disorder through IoT and microservices [42]. However, to the best of our knowledge, there are no ecological approaches that use microservices to perform detailed ML pipelines.

Sensors in combination with ML algorithms have been used to recognize different BADLs such as sitting, standing, walking, running, stair climbing, eating and sleeping [43,44,45,46,47,48,49,50]; and IADLS such as preparing a meal, bathing, using the phone, driving, traveling, and even shopping [46,51]. Some works asses the physical component of frailty in BADLs using wearable devices in combination with ML algorithms (Table 1). Tegou et al. [14] applied several ML algorithms, such as k-Nearest Neighbour (kNN), Random Forests (RF) and Naïve Bayes (NB) to classify the three frailty status based on Fried criteria [4] (non-frail, pre-frail, frail), by measuring the number of transitions between rooms inside the older adult home. Schwenk et al. [15] and Kumar et al. [16] applied Multinomial Logistic Regression (MLR) model to discriminate between the three frailty status, by measuring the gait, balance, and physical activity (PA). Toosizadeh et al. [3] used Ordinal Logistic Regression (OLR) for a frailty detection in 20 s elbow flexion. Greene et al. [17,52] designed a digital assessment based on a Logistic Regression (LR) model for detecting falls, frailty and mobility impairment. Razjouyan et al. [18] applied ML embedded feature selection method for remotely monitoring frailty status, during walking and sleeping BADLs. These studies focused on assessing only physical components of frailty (based on Fried [4]) during physical BADLs (walking, sitting, standing or sleeping), or simple activities such as the elbow flexion or number of transitions from one room to another at home. They neither consider other human dimensions such as cognitive or social functions, nor assess frailty during an IADL (which include these functions). In addition, most of these logistics models did not focus directly on detecting frailty status, instead they looked for the correlation between risk factors (such as falls, balance, physical performance and gait) and the frailty status. Also, the ecological approaches reviewed [16,18] use expensive wearables and questionnaire data such as demographic and clinical data, which do not allow the automation of the data collection. 

Therefore, to the best of our knowledge there are no works that asses the frailty status during the performance of IADLs, combining low-cost sensors with a flexible and extensible system.

## 3. Materials and Methods

This section presents the participants’ sample, the considered variables, the proposed system architecture and the data analysis performed to assess participant’s frailty status, during the shopping IADL performance. First of all, it describes the inclusion and exclusion criteria for recruiting the participants. Secondly, it presents the dependent variable, which is the result of the traditional assessment of frailty by the Fried test. Thirdly, it presents the variables identified through the collected raw data coming from sensory data. Fourthly, it introduces the system architecture designed following the microservices paradigm. Finally, it explains the pipeline workflow designed to apply data analysis considering data collection and labelling; data preprocessing; and machine learning model generation and evaluation.

### 3.1. Sample Description

We conducted a cross-sectional study in three community day centers located in Granada (Spain). We recruited a total of 79 participants (69 women and 10 men) older than 65 years (average age equals 75). The inclusion criteria for this study were: (1) ages ranged from 65 to 90 years old; (2) non severe cognitive decline (using a cut-off score of ≥ 24 points in the Mini-Mental State Examination test [53]); (3) non perceptual alterations, determined by medical diagnosis report. Thus, the participants were included with perceptual alterations corrected with a support device, such as pair of glasses or hearing aid; (4) walking with/without help (cane or walker); (5) community-dwelling older adults. Exclusion criteria: (1) severe mental disorder; (2) severe language alterations; (3) medical instability; (4) pathology in acute stage; (5) hospitalized; (6) serious behavior alterations or motor risks. Our study was approved by the Investigation Ethical Committee of Granada province with reference 0111-N-19 on 31 May 2019 (Andalusian Health Service, Granada, Spain).

### 3.2. Fried Test and Frailty Status Variable

ML models need a training phase and a test phase. The model is trained with labelled data and once the model is created it is tested with different data. Finally, with the model created and tested new data could be automatically classified with one label. We used the score of Fried test [4] to label the data because it is the most accepted test to asses Frailty. This test consists on the assessment of 5 components: slowness in mobility, low energy, low physical activity, muscle weakness, and involuntary weight loss. Depending on the previous factors, elderly people are classified into: “frail” if they score positive in three or more components of the test; “pre-frail” if the score is positive in 2 components and “non-frail” if they do not meet any component. Hence, the Fried test classification (“frail”, “pre-frail” and “non-frail”) will be our dependent variable in the training and test phases of the model.

### 3.3. Wearable Sensors Variables

The independent variables considered come from the raw data of wearable sensors. Previous works used accelerometer, gyroscope and heart rate sensors for monitoring physical activity, health conditions, cognitive, social and other factors [49,54,55,56,57]. We have used similar sensors built-in a low-cost smartwatch, the Samsung Gear S3 [58]. Samsung Gear S3 supports development of software applications on its open access Tizen operating system. This characteristic allows to collect the sensory raw data and to develop software applications in a flexible manner. The built-in sensors we used are: (1) the triaxial accelerometer, which measures changes in the device velocity; (2) the triaxial gyroscope, which detects angular velocity and orientation of the device; (3) and the heart rate sensor, which measures the heart beats per minute. In particular, we collected the raw data from these wearable sensors obtaining 7 variables, detailed in Table 2. The value of these variables was provided in integer number (without a decimal point), or in float (floating-point number, which is a number that has a decimal place).

### 3.4. Microservices System Architecture

Microservice architecture is the next generation of SOA architecture, improving this approach on decoupling, flexibility and extensibility, by sharing services and resources as little as possible. A microservice is a miniature software responsible of a unique and single task (i.e., the minimum level of responsibility and loosely coupled) [30,59,60]. Furthermore, since microservices architecture is based on the concept of share-as-little-as-possible [30], they try to minimize on sharing, whereas SOA tries to maximize on sharing. Consequently, microservices only expose well-defined interfaces. Hence, microservices ensure the maximum decoupling and extensibility, and therefore it is possible to update a microservice independently of the rest of the microservices in an architecture. Indeed, while SOA uses centralized dependencies (such as a centralized data storage), which makes it hard to avoid decoupling, in microservices architectures each microservice manage all of its own dependencies (such as database, key-value store, search index and queue), in order to develop and deploy each microservice independently [30,59]. However, in real scenarios could be necessary to share data between microservices. In our architecture, the data collected by one microservice, need to be stored in a shared database that other microservice could access in order to process the data [60]. 

**Figure 1:**
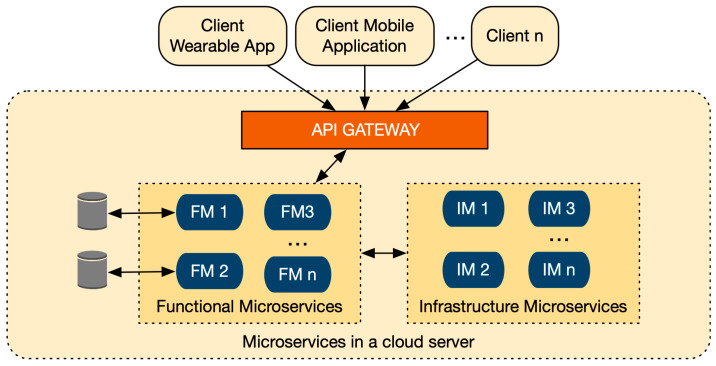
Microservices architecture taxonomy.

Microservices are classified in two groups: functional microservices (FM) and infrastructure microservices (IM) [30]. FMs implement business functions (related with the business domain, e.g., frailty assessment) and can be accessed from external clients (such as wearables, mobile phones and web applications), via an Application Programming Interface (API) gateway. In contrast, IMs implement nonfunctional business tasks such as logging, auditing and monitoring. The main difference between FMs and IMs is that IMs provide a local context and they are not publicly accessible from the outside by client requests, while FMs expose their services publicly. Figure 1 illustrates the microservices taxonomy. In this example, FMs and IMs have been deployed in the cloud, but they can be deployed elsewhere (such as in mobile phones or in wearable devices).

**Figure 2:**
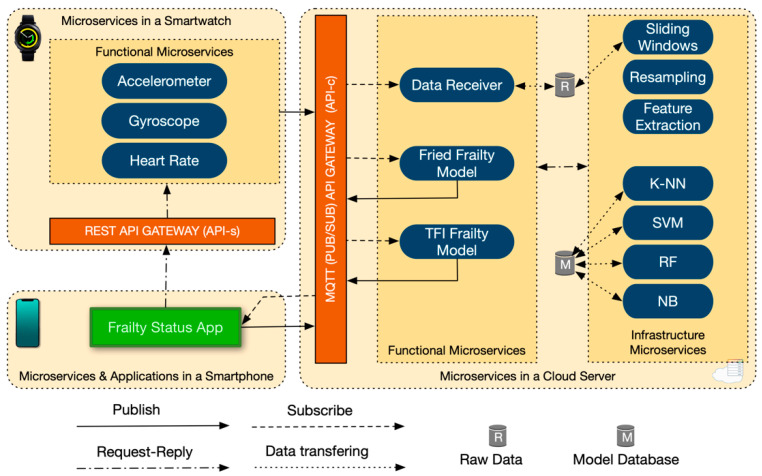
Microservices architecture for frailty assessment.

The functional microservices can communicate with external clients using an API gateway, and with infrastructure microservices via internal requests. For example, in our use case (Figure 2), the application in the mobile phone (Frailty Status App in Figure 2), used by end users, can access the frailty assessment (Fried Frailty model in Figure 2) through the API gateway in the cloud server. The API gateways are the interface components of our architecture, which aggregate microservices. They can distribute information to the external clients (i.e., functional microservices, web and applications) by abstracting the real microservice endpoints. With this abstraction, modifications in the internal microservices are transparent to the clients. For instance, if a microservice is splited into two microservices, clients do not need to change anything [30,59]. 

The API Gateway could be implemented with different protocols, such as REST API [61], CoAP [62], AMQP [63], or MQTT [64]. In particular, we use two API Gateways: a REST API for a one-time communication, and a MQTT for continuous communications. The API gateway in the smartwatch (hereafter, API-s) is a REST API (see Figure 2). API-s setup and start the microservices deployed in the wearable device by invocations from the smartphone. The second API gateway (hereafter, API-c) is deployed in the cloud server and it is based on the MQTT Publish/Subscribe protocol [65] (see Figure 2). API-c supports continuous communication of data between the smartwatch and the cloud. For example, sensor microservices send sensory data and microservices deployed in the cloud stored and processed said data. API-c also supports the communication of events informing the app in the smartphone about the frailty status assessment carried out by a single specific frailty model or even more than one model simultaneously (extensibility), e.g., Fried and TFI. In particular, API-c follows the publish/subscribe communication pattern [65]. The publishers (sensor microservices) push messages (sensory data) through a specific topic (e.g., accelerometer), and the microservices subscribed to that topic (functional microservices in the cloud) receive it. The Publish/subscribe pattern used by the API-c provides decoupling between sender and receiver, allowing asynchronous communications and mobility support. Subscriptions can be established and abandoned dynamically (i.e., Publish/subscribe pattern supports adaptability). Furthermore, MQTT has low overhead, which is essential in sensor environments. In our proposal microservices can publish messages to different topics (communication channels) asynchronously, and others microservices can subscribe to these topics and react accordingly [59].

#### 3.4.1. Microservices Deployed in Wearable Devices

In the wearable device, we deploy only functional microservices, dealing each one with the collection of data from one sensor, namely Accelerometer, Gyroscope and Heart Rate microservices (see Figure 2). The smartphone clients interact with all of the sensor microservices, through the API-s, to setup and start the production of data. Likewise, sensor microservices interact with other microservices deployed in the cloud server through the API-c. In particular, when the Frailty Status App in the smartphone sends the initial setup with the sampling rate to work with and starts the sensors microservices for collecting data (through API-s), the sensor microservices stream the collected data to the API-c in the cloud server. 

Due to the adoption of a microservice architecture, our system is ready to add new wearable sensors in the future (if needed or convenient) for the assessment of frailty or other pathologies. For example, in order to add a new sensor, we need to implement the corresponding sensor microservice and include it into the architecture to collect the sensory data. This sensor microservice should publish data through a new topic using the MQTT protocol (API-c). Likewise, we need to expose the new sensor microservice in the API-s of the smartwatch.

#### 3.4.2. App Deployed in the Smartphone

The purpose of the Frailty Status App deployed in the smartphone is fourfold. First, it requests the sensor microservices to setup and start the data forwarding. Second, it publishes (through API-c) the event that starts one or more frailty model microservices. Third, it subscribes to the topic that allows the reception of events informing about the frailty status of a person. Four, it shows the frailty status to the user (on the mobile application).

Our system can be extended easily with several frailty models to assess frailty and they can even operate simultaneously. To that end, we only need to include the microservice for the new model and create its own topics (one for setup and start process; and another to expose the frailty status). The Frailty Status App will need to subscribe to these new topics. For example, we could include a frailty model which considers different datasets, which could lead to a different model; i.e., using a different sample, for example selecting elderly people with a different age ranges or ethnicities. In fact, when having several frailty models, the Frailty Status App could subscribe to any of the frailty model and status subtopics (e.g., frailty/model1 for starting to work; and frailty/model1/status for getting the frailty assessment result). For example, one Frailty Status App can subscribe to the model-1 subtopic; another Frailty Status App, running in another smartphone, could subscribe to the model-2 subtopic; and even another Frailty Status App could receive results from more than one frailty model at the same time when it is subscribed to the main topic (frailty/), that includes the subtopics.

#### 3.4.3. Microservices Deployed in the Cloud Server

The first microservice we implemented in the cloud server is the functional Data Receiver, which is in charge of receiving and storing the sensory data coming from all the sensor microservices. The Data Receiver receives these raw data without applying any preprocessing. We have also implemented two functional microservices for assessing frailty: Fried Frailty Model microservice (hereafter, Fried microservice) and TFI Frailty Model microservice (hereafter, TFI microservice). Fried and TFI microservices are in charge of the choreography of the corresponding ML pipeline (explained in detail in the next subsection). Both, Fried and TFI microservices assess the frailty status and send the resulting value (“pre-frail”, “frail” or “non-frail”) through the API-c. The pre-built ML models are stored in the Model Database (this offline creation is common in ML domain), in order to use them later by Fried and TFI microservices automatically online. The building process of these models will be explained in Section 3.5.

The infrastructure microservices implement several data analytics techniques. We have implemented three microservices related with preprocessing algorithms (top right of Figure 2): Sliding Windows microservice; Resampling microservice and Feature Extraction microservice, which we will described in detail in Section 3.5.3. We have also implemented as microservices (bottom right of Figure 2), four ML algorithms: k-NN, SVM, RF and NB. These algorithms require the pre-built frailty models stored in Model Database (see Figure 2) in order to calculate the frailty assessment (”frail”, “pre-frail” or “non-frail”). They require as well, the preprocessed data from the previous microservices (sliding windows, resampling and Feature Extraction microservices). 

Adding a new frailty model to our system, requires the following: (1) to pre-build the new ML model, and to store it in the Model Database; (2) to create the new frailty microservice for the new model, together with its own subtopics (frailty/newmodel; and frailty/newmodel/status) and ML pipeline setup; and (3) if we need a new ML algorithm not yet implemented, such as Artificial Neural Network algorithm, we need to create it as an infrastructure microservice.

#### 3.4.4. Workflow

**Figure 3:**
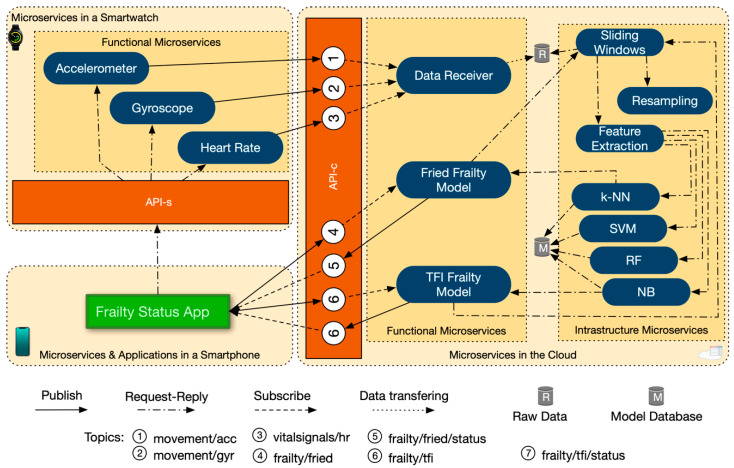
Workflow communication details of the microservice architecture for frailty assessment.

Figure 3 shows the workflow communication during the assessment of frailty. In this section, we show the potential of our architecture in terms of extension of future business functionalities, illustrating a scenario with two frailty models—Fried and TFI. In particular, we will explain in detail the workflow communication between microservices and app through the APIs and internal requests and events.

First, the Frailty Status App sends a REST POST request to start data forwarding from each sensor microservice available in the smartwatch (in particular, the Accelerometer, Gyroscope and Heart Rate microservices). As a response, all the sensor microservices publish their raw data into the API-c gateway to the corresponding topics: “movement/acc”, “movement/gyr”, and “vitalsignals/hr”, respectively. Then, the Frailty Status App publishes an event associated to the topic “frailty/fried” with a value of “1”, and another event “frailty/tfi” with a value of “1”, in order to start the Fried and TFI microservices, respectively. When the app wants to stop the model microservices it will send the same event with a value of “0”.

Second, the Data Receiver microservice, which is subscribed to all of sensory data topics (“movement/acc”, “movement/gyr”, and “vitalsignals/hr”), receives the raw data from the sensor microservices, and stores the data. Simultaneously, the Fried and TFI microservices, which are subscribed to the topics “frailty/fried” and “frailty/tfi”, respectively, and start their choreographies. These choreographies consist of a list of sequential ML pipeline microservices with their respective parameters, which are sent to the first IM in the pipeline (in our case Sliding Windows microservice). For example, for Fried microservice the choreography consist of: (1) sensor microservices to use (accelerometer, gyroscope and heart rate); (2) data sampling rate (25 Hz); (3) sliding windows size (0.5 s); (4) subset of feature extractions (mean, standard deviation, minimum and maximum values, kurtosis, skewness and energy—explained in Section 3.5); (5) pre-built frailty model (e.g., pre-built Fried model database path); (6) the ML algorithm to make the frailty assessment (e.g., k-NN); (7) and the returning Frailty model microservice where to send the frailty status assessed in step 6 (e.g., Fried microservice).

Third, the ML pipeline, which will be described in detail in next subsection, starts. The Sliding Windows microservice receives the ML pipeline setting, and each microservice of the pipeline is activated sequentially by the previous one. Finally, the ML microservices, which are the last of the pipeline, send the resulting frailty status to the corresponding Fried or TFI microservice.

Fourth, the Fried microservices receive the frailty status predicted by the ML microservice and publish the result of the frailty assessment—“non-frail”, “pre-frail’ or “frail”—to the topic “frailty/fried/status”. Likewise, TFI microservice publishes the result to the topic “frailty/tfi/status”. Then, Frailty Status App on the smartphone receive this result and notifies the user.

### 3.5. The Data Analysis Pipeline to Build a Predictive Model for Frailty Assessment

**Figure 4:**
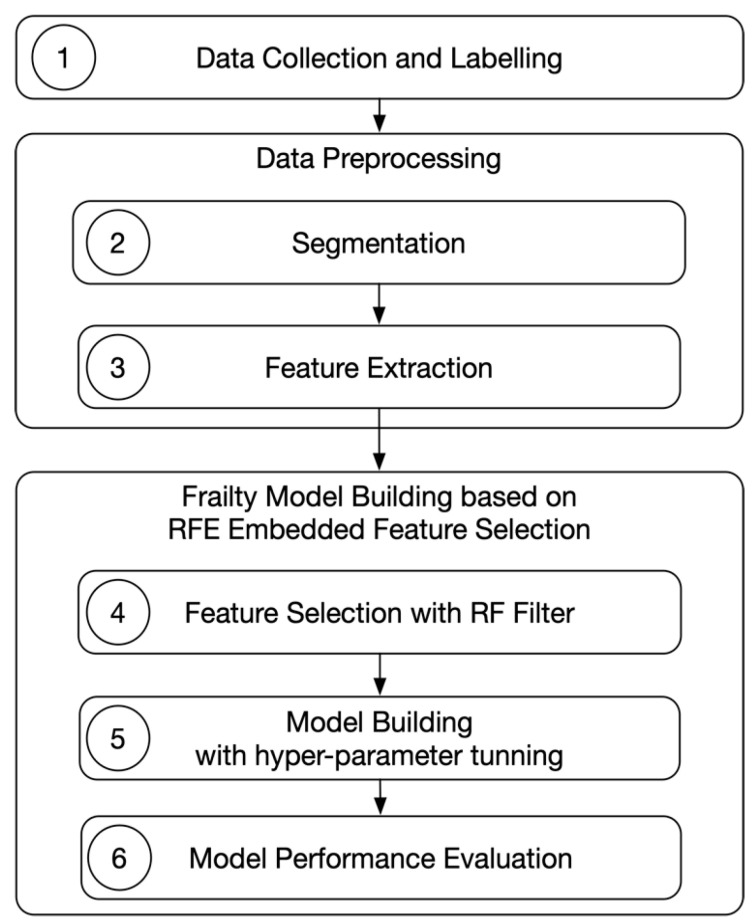
Data analysis pipeline for frailty status assessment.

In order to build a frailty model, we have designed a data analysis pipeline, which includes three main phases: (1) data collection and data labelling; (2) data preprocessing; (3) and frailty model building with ML techniques. In the first phase, we collect the data and label it in order to apply machine learning algorithms in supervised manner, which need labelled data. Our health experts classify each older adult with a label (“frail”, “pre-frail” and “non-frail”) according to the traditional assessment of frailty based on Fried test [4]. This label becomes the dependent variable for assessing frailty status. In the second phase we preprocess the data focusing on the enhancement of the model performance. Usually, preprocessing techniques include segmentation, feature extraction and dimensionality reduction (i.e., feature selection) [43]. In our case, the preprocessing techniques applied consist in the feature extraction combined with segmentation, and feature selection to reduce these features extracted. In the third phase, we build a predictive model using the labelled and preprocessed data. We build the model outside the system architecture as we mentioned above, but we sored the model in a database accessible by our Fried microservice. Figure 4 shows all the phases and steps inside the pipeline, which will be described in detail bellow.

#### 3.5.1. Data Collection and Labelling Process

In order to make the frailty assessment, we collected the results of the participants’ frailty status based on Fried classification. Thus, we had labelled all of these participants with their corresponding frailty status (“frail”, “pre-frail” and “non-frail”).

Participants were asked to buy a specific product in the nearest supermarket following the next protocol. The participant starts in a sitting position on a chair without armrests, with €1 coin and wearing the Gear S3 smartwatch in the non-dominant hand. The supervisor, who is responsible of the experiment supervision, starts the Frailty Status App to start capturing wearable sensory data. The participant stands up and walks to the supermarket; looks for and pick up a 1 kg package of salt; goes to the check out and pays; comes back to the starting point; and sits down on the chair. The supervisor stops the collecting of data with the Frailty Status App.

The performance of the shopping activity was divided into several tasks or sub-activities doing an activity analysis using the Occupational Therapy Practice Framework [66]. During this whole process, the evaluator labeled the different tasks of the shopping activity during its execution: (1) sitting; (2) standing; (3) walking to the supermarket; (4) in the supermarket; (5) looking for the product to purchase; (6) picking the product; (7) going to the checkout; (8) in the checkout; (9) paying; (10) go to the exit; (11) in the outside; (12) coming back; (13) standing at the start point; (14) and sitting back. This labelling was performed by observation, and with the help of an app connected to the smartwatch.

The smartwatch transmits (offline) the data collected to the smartphone (to the Frailty Status App) via Bluetooth. The sampling rates used in the data collection is normally greater than 10 Hz, and in most cases ~20 Hz, as similar studies in the field of activity recognition suggest [50,51,67]. In particular, we recorded data at 25 Hz as in Genovese et al. [55] and close to the 20 Hz used by Garcia-Ceja et al. [51]. Nonetheless, we have performed several experiments with different sampling rates between 10Hz and 100 Hz, obtaining similar results. Moreover, we excluded anomalies in heart rate values based on the formula: HR maximum = 220 − participant age [68,69]. Furthermore, as we recruited participants in three community-centers, the distance from the center to the supermarket were different (two centers: 50 m; and the third 100 m). This fact does not carry any inconvenience as the activities are labelled (i.e., when the participant starts and end walking to the supermarket) and the distance for each participant is also recorded. 

#### 3.5.2. Data Preprocessing

In order to get accurate results assessing frailty [70], we performed two well-known data preprocessing techniques sequentially: (1) segmentation of raw data (without preprocessing) with 50% overlapped windows strategy; (2) feature engineering, for every window, in order to extract relevant variables.

##### Segmentation

We implemented 50% overlapped sliding windows approach to segment the data, as in previous works [50,67,71]. This is a common technique used with wearable sensory data, which implies an increase of the sample size due to the overlap, and therefore the re-used of the data. Hence, this technique improves the accuracy of the results [43]. We test different window sizes to get which of them reports the best model performance: 0.5 s; 1 s; 1.5 s; 2 s; and 2.5 s.

##### Feature Extraction

We also apply feature selection, as in other similar works [12,49,50,72,73]. In particular, we extract eight statistical features for every wearable variable (we identified 7 wearable variables in Table 2) and for each window. Related with time-domain, the extracted features are: mean, standard deviation (SD), skewness (the probability distribution asymmetry), kurtosis (the probability distribution, a.k.a. tailedness), maximum, minimum and amplitude (the absolute difference between the maximum and minimum). The only frequency-domain feature extracted is the energy, which is the sum of the squared Fast Fourier Transform (FFT) components [74,75]. In order to normalize the energy feature, it was divided by each window size ($\left|w\right|$). Let x_1_, x_2_, …, x_n_ be the *i*-th FFT components for the windows w_1_, w_2_, …, w_m_, then Energy [74] expression is:

(1) $$\mathrm{Energy}=\frac{\sum_{i=1}^{\left|w\right|}{\left|x_{i}\right|}^{2}}{\left|w\right|}$$

#### 3.5.3. Frailty Model

##### Feature Selection

Feature selection is a technique that selects only the most relevant features to train the model and reduces the dimensionality of the dataset. As we could train the model with only a selection of features: the model will train faster; the complexity of a model is lower and therefore it makes it easier to interpret; the accuracy of a model could improve if the right subset is chosen; and the overfitting is also lower. Feature selection methods apply statistics to identify what are the most important features and remove the redundant features. A feature is redundant when another relevant feature exists with a similar power of prediction [76]. The feature selection techniques are the filter, wrapper and embedded methods [16,77]. Filter methods identify the relevant features based on statistics, without considering a machine learning classifier to build a model. Wrappers use a subset of features to measure the performance of a machine learning classifier; and then they iteratively add or remove features to the subset according to the results, until they reach an optimum. Embedded methods include feature selection as part of the machine learning model building. Therefore, both wrapper and embedded methods are specific to the machine learning algorithm used.

In our case, we have used embedded methods (such as in [18]), hence using feature selection in the building process of the frailty assessment model. Our embedded method consists in the combination of a filter method (based on Random Forest to rank features by their importance) with the Recurrent Feature Elimination (RFE) strategy [78] to build (also called train) one ML model per feature selected. First, RF sort the features by their relevance in descending order, then RFE starts considering all of the sorted feature set. Then, we build the model with that sorted feature subset and evaluate different performance metrics (we will detail these metrics in the next subsection). In each iteration, we eliminated the least relevant variable (or redundant); recalculate the importance rank of the new subset with RF filter; create a model with this subset; and evaluate the frailty model performance. At the end, we select the model with the best performance and identify what are the resulting features subset. Considering that the total wearable variables coming from the raw data are 7 (see Table 2) and that from each variable we extract 8 features, our initial set of features is 56.

##### Frailty Model Building

To build an accurate machine learning model it is necessary to tune its hyper-parameters, considered high level concepts of the model. For instance, some hyper-parameters are complexity, capacity to learn, number of leaves in trees of RF, or kernels in SVM [79]. As described in the related work, several machine learning algorithms have been applied successfully to assess physical frailty, such as k-NN, RF and NB. We used these algorithms and Support Vector Machines (SVM), which is widely used for ADL recognition. Therefore, we tried them all, and identify which one of them reported the best performance for our use case. We tested these algorithms, with R programming language, tuning its specific hyper-parameters based on related studies [45,49,80]:

- k-NN: (1) k: {1, 3, 5, ..., square of number of rows} (only odd numbers).
- SVM: (1) cost function: {0.1, 1, 10, 100}; (2) gamma value: {0.5, 1, 2}; (3) and, kernel type: {“radial”, “polynomial”, “linear”, “sigmoid”}.
- RF: (1) number of trees: {10, 100, 200, 500, 1000}; (2) number of variables randomly sampled: {10, 25, 50}.
- NB: (1) use of kernel: {True, False}; (2) use of poisson: {True, False}.

Regarding the computational load, it is known that in the creation of the model (training phase), some algorithms are slower (SVM and RF) than others (k-NN and NB). However, this process is performed offline and it does not require rapid responsiveness. The complexity of the offline building process for every ML algorithm is as follows. Let n be the training size, m the number of features, n_trees_ the number of trees (for RF algorithm), then the computational complexity is:

- k-NN: O(nm)
- SVM: O(n^2^m + n^3^)
- RF: O(n^2^mn_trees_)
- NB: O(nm)

However, once we have our model built, to know the prediction of a particular record (i.e., to assess frailty of a person) the computational complexity is reduced. Specifically, the complexity of each ML algorithm is:

- k-NN: O(nm)
- SVM: O(n_sv_m), where n_sv_ is the number of support vectors, which is the resulting points of the SVM model, close to the decision boundary.
- RF: O(n_trees_m)
- NB: O(m)

##### Model Performance Evaluation

In order to validate our ML model, we used 5-fold stratified cross-validation [3,14], in which the dataset was randomly divided into k = 5 equal parts keeping the proportion of samples of the three frailty labels (“frail”, “pre-frail” and “non-frail”). At each k-th iteration, the k-1 partitions were used to train (build) the model, and the left-out partition (hidden data for the trained model) was used for validating the model. The performance metrics for this validation were: accuracy, f1-score, sensitivity (true positive rate) and specificity (true negative rate). These metrics are related with these concepts: (1) true positive (TP) condition, which is the number of true positive cases in the data and rightly predicted as positive by model; (2) true negative (TN) condition, which is the total number of true negative cases and rightly predicted as negative; (3) false positive (FP) condition, which is the total number of positive cases classified wrongly as negatives; (4) and, false negative (FN) condition, which is the total number of negative cases but classified wrongly as positives. Taking into account these considerations, the expressions of the four metrics used are the following:

Accuracy = (TP + TN) / (TP + TN + FP + FN)(2)

Sensitivity = TP / (TP + FN)(3)

Specificity = TN / (TN + FP)(4)

F1-Score = 2TP / (2TP + FP + FN)(5)

## 4. Results

Before using the system, we performed a system validation, with only five participants, in which we validated the technological solution, as well as the usability and acceptance, following a user-centered design approach. After this preliminary validation, we were ready to validate the complete frailty assessment system with a larger sample of 78 participants.

### 4.1. System Validation Results

We carried out a preliminary study [24] as a proof of concept for the technical validation of the system architecture and the usability and acceptance by end-users, i.e., experts and the older adults. Debes et al. [46] suggested that the acceptance of wearables for Health assessment can be increased with a user-centered design, that guarantees privacy and transparence. Therefore, we not only design at the beginning for elderly people, but also adapt the system to them. Taking this into account, we performed this proof of concept in order not only to validate the technical proposal, but also to adapt the system to the users.

**Figure 5:**
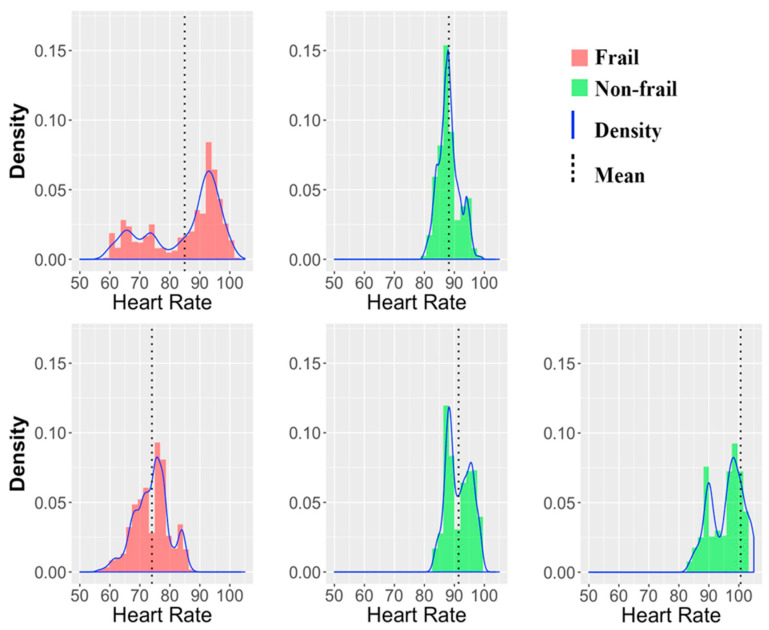
Comparison between frail and non-frail individuals by heart rate [24].

In order to validate the technological solution, older adults were assessed in a real environment (performing a shopping activity at the supermarket). The participants recruited for the proof of concept were five adults coming from a community center in Granada (Spain); three of them were women (one frail; and two non-frail); and two were men (one frail and one non-frail). The average age was 84 years old. All of them performed the shopping IADL, as explain in Section 3.5.1, while we collect sensory data using our system architecture. After that, we performed a preliminary Exploratory Data Analysis (EDA) with the collected data revealing that heart rate data could be a relevant feature to distinguish between non-frail and frail participants (Figure 5).

In particular, frail individuals reported a wider range of heart rate over the non-frail individuals. Specifically, heart rate values in frail individuals move between 50 and 90/100; whereas, in non-frail individuals the values move only between 80 and 100. In addition, the mean heart rate values were minor than 88 in frail participants, and greater than 88 in non-frail participants. However, these observations are a proof of concept of the system, and the sample is not representative (only 5 participants) for inferring that wider range of heart rate is a feature exclusive of non-frail individuals. Nonetheless, this proof of concept represents that our system is technically validated, and ready to perform a frailty assessment with a larger dataset.

We also validated our system in terms of usability, acceptance. The supervisor of this preliminary experiment observed the participants and interviewed them about this activity. Participants answered whether the wearable affected their comfort and mobility. The main findings are in line to those of Ehmen et al. [81]: (1) older adults with low sensorimotor abilities can use our system; (2) our system requires little time of use and it is transparent for older adults, because they only have to wear the smartwatch; (3) our system is easy to use for older adults, they do not require any technological competence; and (4) our system does not require extra personnel to train the users or to use the system. In conclusion, all of the participants reported a satisfactory experience. They said that the system was non-intrusive. Participants also reported that: the wearable is easy to wear; the app is easy to learn; and the collected data are secured because they are anonymized.

Therefore, the technological solution was viable, and the system was adapted to the end users (user design centered) without the need to any further tuning. Hence, we started the assessment of the frailty with a larger sample.

### 4.2. Frailty Model Results

In this section, we present the results of the frailty model building: first, the recount of frailty classes of the recruited sample, with/without considering shopping phases; second, a summary of the ML algorithms used and total features explored; third, the best ML model built and its metrics; and finally, a comparative about how shopping IADL influences in the model performance for frailty assessment.

Wearable sensory data of 79 participants (69 women; 10 men) were collected. However, the exploratory data analysis reported one male with some missing values, thus we discarded this record. Then, the final sample consisted in 78 participants, 12 of them classified as “frail”, 47 “pre-frail” and 19 as “non-frail”. In addition, we labelled the global shopping activity into 14 shopping tasks or sub-activities (as described in the Materials and Methods section), with the help of an occupational therapists’ analysis to define the phases. The final distribution sample is: (1) 168 phases for “non-frail” individuals; (2) 658 phases for “pre-frail” individuals; (3) and 266 phases for “frail” individuals. In total 1092 samples.

**Figure 6:**
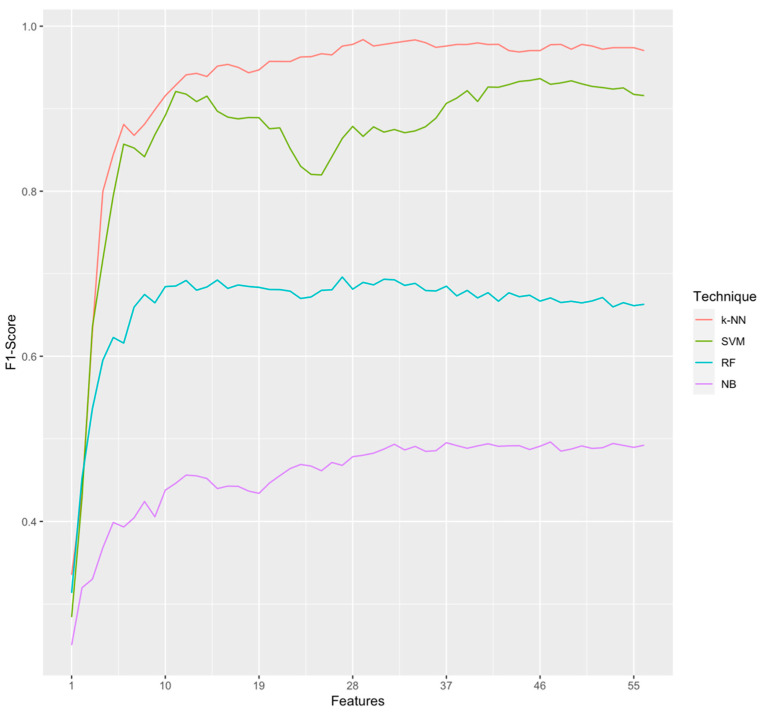
Performance of different machine learning algorithms by RFE embedded feature selection.

We built four frailty classification models using the machine learning algorithms k-NN, SVM, RF and NB, following the embedded feature selection method (with RF filter to rank features) and using RFE strategy. Then, we used 5-fold stratified cross-validation to evaluate all of models built. Figure 6 shows the metric F1-score over features from 1 to 56 of total amount of features extracted.

Table 3 shows the results. The k-NN model reported the best performance with k = 1 (i.e., 1-NN) at 25 Hz, 0.5 s windows size and considering only 29 features followed closely by SVM. The best accuracy was 0.99, i.e., it classifies correctly the frailty status in most than 99% of the participants recruited, using only 29 features extracted from the wearable sensors. In 1-NN, F1-score was 0.98. Sensitivity was 0.97 and specificity was 0.99. In addition, we found that for 1-NN (see Table 4), the best frailty status detected was “pre-frail”, followed by “non-frail” and, then, “frail”, giving priority to sensitivity metrics rather than specificity, which is preferred in health problems.

**Figure 7:**
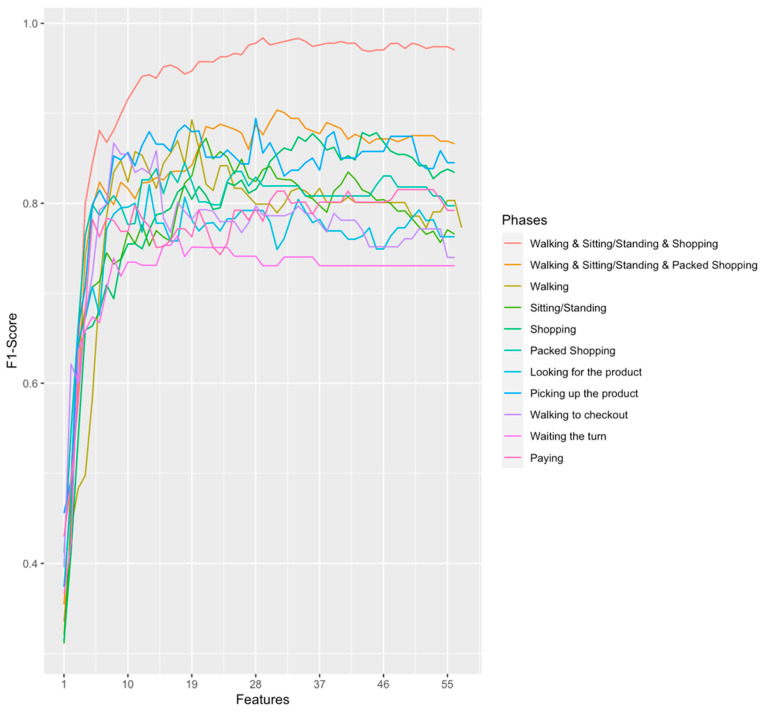
Performance of 1-NN by RFE embedded feature selection over shopping phases. Walking: (1) walking to the supermarket; (2) coming back. Sitting/Standing: (1) sitting; (2) standing; (3) standing at start point; (4) and sitting back. Shopping: (1) in the supermarket; (2) looking for the product to purchase; (3) picking the product; (4) going to the checkout; (5) in the checkout; (6) paying; (7) go to the exit; (8) in the outside. Packed Shopping: all phases of Shopping but packed in only one phase.

Furthermore, we used these findings in order to test 1-NN to know which are the most relevant shopping phases for the final assessment of frailty status. To that end, we perform several experiments with different phases or grouping of phases in them. For example, automatic detection of the shopping phases inside the supermarket could pose a challenge. Therefore, we performed an experiment consisting in grouping all these stages in one single phase in order to compare the impact of packaging phases or not. This package process (packed shopping) consisted in reducing the number of all shopping phases samples by computing the arithmetic mean of all the shopping phases values (row 4 in Table 5). As expected, the results indicate that the performance is worst, by 2.6%, if we consider the shopping phases packaged (row 6 in Table 6) than if we considered one single phase separately (rows 5 in Table 6). Figure 7 shows these results and additionally, we can see how the F1-score becomes stable after 20 features approximately for most of the phases. In particular, for the phase “Walking & Sitting/Standing & Shopping” we can see that it increases linearly with the number of features until 15 features, and then improves slowly until 29 features where it reaches its maximum. Furthermore, the most influent shopping phase is “looking for the product” with a 93.2% accuracy, followed by “walking to the checkout”.

## 5. Discussion

We have created a platform based on microservices which allows the generalization, extension and reuse of our system functionalities. In particular, we can easily include new sensors coming from different wearables, such as smartwatches, wristbands, bracelets and headbands. Likewise, we can reuse or include new microservices to predict other pathologies (e.g., the dependency status).

We found that the shopping activity (IADL) is equally important than individual BADLs in the assessment of frailty, but the combination of both improves the accuracy. The prediction of frailty status has a similar accuracy considering only the shopping IADL (row 5 in Table 6) or considering only the BADLs (rows 3 and 4 of Table 6) such as sitting, standing and walking outside the supermarket. However, the addition of shopping IADL data to sitting, standing and walking BADLs data (see row 1 in Table 6) increases the accuracy in 5% (from rows 3 and 4 to row 1 in Table 6). This combination reaches the best accuracy of 99.2% using k-NN algorithm with k = 1 (1-NN). Then, since k-NN algorithm works with Euclidean distance, and provides better results than other ML algorithms tested, this suggests our classes (three frailty status) are quite separable. Furthermore, the most influent shopping phase is “looking for the product”, with 93.2% accuracy followed by “walking to the checkout”. These results could be due to the fact that both phases involve the physical activity of walking, which is the BADL with the best results. The worst accuracy was achieved by the “waiting in turn” phase, which probably is due to the fact that it does not involve any physical or cognitive activity and the sensors could not discerned frailty form non-frailty older adults. The second worst results (although still a good results) was provided by the “paying” phase. This result is probably due to the fact that paying involves cognitive and social factors, that we do not considered when we labelled the data with Fried test. Thus, we will explore in the future the use of sensors, such as Electrodermal Activity (EDA), which could sense physiological signals which are known to be directly correlated to cognitive and social functions; as well as labelling the data with other frailty tests which considered social and cognitive functions. Nonetheless, our findings confirm the relationship between the IADLs performance with the frailty status of older adults [9] and state that the combination of BADLs and IADLs improve the accuracy.

Our findings with “shopping packed” are relevant, because the detection of a person inside of a supermarket (and therefore performing the “shopping packed” phase), could be easily detected, for example, using a GPS location sensor or some IADL recognition methods available in the literature. Whereas the detection of each phase inside the supermarket could be a challenge or require more resources, such as fixing sensors inside the supermarket.

Our frailty model outperforms the previous works. In particular, it outperforms the most relevant study comparable to ours we found [14], as mentioned in Section 2, which has an accuracy of 82.33% and sensitivity of 83.83% classifying the three classes of frailty status (frail, pre-frail and non-frail), but using only BADLs. Also, our model has an accuracy 1.26% higher than the other experiment performed by Tegou et al. [14], which divided the sample in two classes (group 1: dentification of frail participants; group 2: non-frail and prefrail participants together as a single class). In addition, we outperform [18] from 85% to 99% accuracy, which used embedded feature selection approach as our proposal.

Our proposal is novel because it is ecological, low-cost, wearable-based to automatize the data collection non-intrusively—applying ML techniques to classify the three classes of frailty syndrome—microservices based architecture for healthcare systems. Furthermore, our proposal outperformed similar studies, which measure Frailty with ML, but do not have all the above characteristics.

This paper may have some limitations. Firstly, the recruited sample had an unequal gender distribution, which is common in this kind of experiments with elder people. Likewise, the sample is imbalance, due to the bigger number of “pre-frail” people in this age range and able to perform IADLs, than the number of “frail” or “non-frail”.

The benefits of our results consist on the automatic assessment of frailty status during IADLs, which involves an ecological approach. This could reduce health professionals’ time, health costs, and could be the basis for a preventive intervention to reduce frailty. Our proposal is ecological because, we can measure frailty of older adults in their own environment (their daily living), without disturbing them, in a non-intrusive and transparent manner. A consequence of this ecological point of view, is the reduction of costs and time in the assessment of frailty, in comparison with traditional frailty tests. Furthermore, due to the adoption of microservices approach in our system architecture we can take advantage of all of its properties, such as extensibility and reusability. For that reason, we are able to extend the system with different frailty model microservices or including different sensors. Moreover, our frailty model contributes to an early detection of frailty status, which is a key point for prevention [4,5], allowing health professionals to make early decisions for performing specific interventions.

## 6. Conclusions and Future Work

In this paper, we have presented a novel proposal for e-health, which is a platform based on microservices for assessing frailty status of older adults, ecologically during the performance of an IADL, such as shopping, which involves physical, cognitive and social human functions. To that end, we designed a microservice architecture to support sensory data collection from wearables, and ML analytics. Hence, our proposal take advantage of the microservices characteristics, such as extensibility, reusability and scalability. The main potential of the microservices architecture is that it could be extended with little effort. For example, we could easily include new data sources, new sensors, new business functionalities, or new frailty models based on different assessment methods. Our ML model has an accuracy of 99.2%, a 98.4% F1-score, a 97.7% sensitivity and a 99.5% specificity, outperforming those of the previous works. Our system is ecological, because it does not disturb the daily living of elderly people. Our frailty assessment is performed in a transparent and non-intrusive way, thanks to the use of mobile computing technologies. 

Our proposed model can help healthcare professionals in the early detection of frailty, reducing costs and time. It can help clinicians in the early detection of frailty, because the monitorization could be done automatically and continuously, even before any symptoms appear. Hence, clinicians could design personalized intervention plans to prevent or revert frailty. Traditional frailty assessments require to perform several tests of gait, balance, physical activities and self-report questionnaires. Thus, healthcare services could adopt our solution, because its implementation implies a more objective assessment than the traditional tests, which involves questions about the feelings of your personal health and independence. Our solution also implies a time reduction and a decrease of cost with respect to the conventional assessments. 

## Figures and Tables

**Table 1:** Review of previous works related to assessing frailty with wearables and ML.

Work	Aim	Eco	Data Sources	System	Frailty Status	Best ML
[14]	To assess frailty by a system based on Bluetooth RSSI fingerprints using beacons, collecting data derived from transitions among rooms	Yes. Transitions between rooms.	Smartphone Beacons (low-cost)	RSS	Three ^2^ and two ^3^	RF ^2^: Accuracy: 82.33% Sensibilty: 83.83% RF ^3^: Accuracy: 97.92% Sensibilty: 94.2%
[15]	To discriminate between frailty status with gait, balance or during a physical activity.	No	LEGSys ^1^ ($10,000) BalanSens ^1^ ($4450)	None	Three ^2^	MLR: AUC: 85.7%
[16]	To implement a wearable to characterize the quantity and quality of everyday walking, and to establish associations between gait impairment and frailty.	Yes. Walking ADL during 2 days	PAMSys ^1^ Demographic Clinical	None	Two ^4^	MLR: Accuracy: 77.7% Sensibilty: 76.8% Specificity: 80%
[3]	To assess frailty by a wearable during the flexibility of upper-extremity movements.	No	Gyroscope ^1^	None	Three ^2^	OLR: Accuracy: 69%
[17,52]	To design a digital assessment protocol and algorithm for prediction of falls, frailty and mobility impairment.	No	Shimmer ($495) Demographic Clinical	None	Two ^4^	LR: Accuracy: 72.8% Sensibilty: 72.99%
[18]	To remotely monitor the frailty status using an accelerometer.	Yes. Walking & Sleeping ADLs during 2 days	PAMSys ^1^ Demographic Clinical	None	Two ^5^	EFS: Accuracy: 84.7% Sensibilty: 91.8% Specificity: 81.4%

Eco: Is it ecological? Is it measuring frailty in the users’ daily living environment? RSS: Received Signal Strength; RSSI: Received Signal Strength Indicator; AUC: Area under the curve. RF: Random Forest; MLR: Multinomial Logistic Regression; OLR: Ordinal Logistic Regression; EFS: Embedded Feature Selection. ^1^ BioSensics LLC manufacturs non low-cost wearables devices. ^2^ Considered Fried 3 classes: non-frail, pre-frail, frail status. ^3^ Considered Fried 2 classes: identification of frail participants against non-frail and prefrail participants together as a single class. ^4^ Considered Fried 2 classes: identification of non-frail participants against pre-frail and frail participants together as a single class. ^5^ Considered Fried 2 classes: identification of pre-frail participants against non-frail and frail participants together as a single class.

**Table 2:** Wearable sensors variables from raw data.

Variable Description	Type
Accelerometer *X*-axis value	Float
Accelerometer *Y*-axis value	Float
Accelerometer *Z*-axis value	Float
Gyroscope *X*-axis value	Float
Gyroscope *Y*-axis value	Float
Gyroscope *Z*-axis value	Float
Heart Rate value	Integer

**Table 3:** Performance of different Machine Learning algorithms to assess frailty status.

Algorithm	Features	Accuracy	F1-Score	Sensitivity	Specificity
k-NN ^1^	29	**0.9917641**	**0.9837171**	**0.9764216**	**0.9947197**
SVM	46	0.9670102	0.9364576	0.9108271	0.9779242
RF	27	0.8461648	0.6960141	0.6244533	0.8733734
NB	47	0.6621256	0.4960688	0.4353061	0.7659894

^1^ k-NN reached the best performance with k = 1 (1-NN). In bold letters, the best performance results.

**Table 4:** Performance of 1-NN for the three frailty status.

Frailty Status	Sensitivity	Specificity
Frail	0.9375	0.9946237
Pre-frail	**0.9851852**	**0.9879518**
Non-frail	0.962963	0.9939024

In bold letters, the best performance results.

**Table 5:** Experiment names and phases (tasks or sub-activities) of shopping considered.

Experiment (Phases)	Tasks or Sub-activities
Walking	(1) Walking to the supermarket (2) Coming back
Sitting/Standing	(1) Sitting (2) Standing (3) Standing at start point (4) Sitting back.
Shopping	(1) Participant is in the supermarket (2) Looking for the product to purchase (3) Picking the product (4) Going to the checkout (5) In the checkout (6) Paying (7) Go to the exit (8) In the outside
Packed Shopping	(1) Same phases as the shopping experiment but considered as a unique phase by computing the arithmetic mean of the values.

**Table 6:** Performance of 1-NN in different experiment phases.

Algorithm	Features	Accuracy	F1-Score	Sensitivity	Specificity
Walking ^1^ & Sitting/Standing ^2^ & Shopping ^3^	29	**0.9917641**	**0.9837171**	**0.9764216**	**0.9947197**
Walking ^1^ & Sitting/Standing ^2^ Packed Shopping ^4^	31	0.9503722	0.9036798	0.8792540	0.9705433
Walking ^1^	19	0.9425269	0.8927655	0.8656145	0.9650742
Sitting/Standing ^2^	21	0.9325653	0.8722884	0.8430592	0.9594234
Shopping ^3^	42	0.9359852	0.8785180	0.8397436	0.9588485
Packed Shopping ^4^	18	0.9091168	0.8450966	0.8034157	0.9488836
Looking for the product	28	**0.9322537**	**0.8944003**	**0.8754377**	**0.9671364**
Picking up the product	15	0.8940171	0.8218771	0.7855828	0.9441125
Walking to checkout	8	0.9245014	0.8669296	0.8295743	0.9552347
Waiting for their turn	17	0.851007	0.7638345	0.7256926	0.9190001
Paying	48	0.8863248	0.8151564	0.7741049	0.9367538

^1^ Walking: (1) walking to the supermarket; (2) coming back; ^2^ Sitting/Standing: (1) sitting; (2) standing; (3) standing at start point; (4) and sitting back. ^3^ Shopping: (1) in the supermarket; (2) looking for the product to purchase; (3) picking the product; (4) going to the checkout; (5) in the checkout; (6) paying; (7) go to the exit; (8) in the outside. ^4^ Packed Shopping: all phases of Shopping ^3^, but packed in only one phase. In bold letters, the best performance results.
